# Structural Development

**Published:** 1897-12

**Authors:** G. N. Peirce

**Affiliations:** Philadelphia, PA.


### Structural Development.

BY G. N. PEIRCE, D.D.S., PHILADELPHIA, PA.

Abstract of paper read at American Dental Association, August, 1897.

In connection with Section VII, Dr. W. C. Barrett, chairman, placed on exhibition an immense collection of human and comparative odontology which Dr. Peirce described as presenting an array of landmarks rarely seen in a meeting or an association of professional individuals, unless of a strictly scientific character. He said: Each one of these representations has a history, and from each one, the nearest akin but more complex, has derived some organ, trait or peculiarity essential to his being, and this is true from the most simple to the most complex—from the Batrachian to Man.

The organs which are held in common indicate the road that has been traveled from the most primitive to the most highly specialized. The general plan of construction was a unit: the divergencies in response to the necessity of adaptation to environment, intensified by inheritance. It is a recognized fact that any change in the circumstances, that is, in the sum total of influences affecting any organism, will be likely to work some alteration in that organism or in its descendants, or in both.

The teeth are of more interest to the dental profession than any other set of organs considered separately, and yet these organs subserving nutrition cannot be studied without a marked correspondence with organs being noted.

A suggestive query is, " Are the teeth, with other organs of living beings, adaptive or non-adaptive? Are they machines especially fitted to meet the demands of their environment, or are they not?" The question, "Did the occasion for its use follow the appearance of the structure, or did the need for the structure precede its appearance?" is answered in the words of the late Prof. Cope, "Animals and plants are dependant for existence on their environment;" "Changes in environment occur without any preparation for them on the part of living things;" "The influence of environment is brought to bear on life as it is or has been and special adaptations to it on their part must follow, not precede, changes of climate, topography, population, etc." Another important consideration is the well-known and recognized influence of use, motion on nutrition—the law of use and effort—kinetogenesis.

In the study of teeth it is not difficult to recognize that there are certain central or primitive types, from which it is easy to derive other related forms of dentition by simple addition, subtraction or modification of parts already possessed. The so-called teeth of the invertebrates are enderonic in origin—an oral armature with nothing in common with the teeth of vertebrates except their similar functions. The teeth of the vertebrates are enderonic in origin, in shape and function ranging from a simple cone for seizing and retaining, to the incisors for cutting, canines and carniverous molars for tearing or lacerating, and tubercular for crushing, and the complex grinding molar of the omniverous and herbiverous for the trituration of food.

